# Sex of Walker Influences Scent-marking Behavior of Shelter Dogs

**DOI:** 10.3390/ani10040632

**Published:** 2020-04-07

**Authors:** Betty McGuire, Kentner Fry, Destiny Orantes, Logan Underkofler, Stephen Parry

**Affiliations:** 1 Department of Ecology and Evolutionary Biology, Cornell University, Ithaca, NY 14853, USA; kf287@cornell.edu (K.F.); dmo64@cornell.edu (D.O.); 2 143 Carter Creek Road, Newfield, NY 14867, USA; underkof@gmail.com; 3 Cornell Statistical Consulting Unit, Cornell University, Ithaca, NY 14853, USA; sp2332@cornell.edu

**Keywords:** dog, scent marking, urination, urinary posture, defecation, ground scratching, animal shelter, human-animal interactions

## Abstract

**Simple Summary:**

In diverse settings, human presence and handling influence the behavior and physiology of other animals, often causing increased vigilance and stress, especially if the human is unfamiliar. Domestic dogs are unusual in that human interaction often reduces stress and behavioral signs of stress. Nevertheless, there is some evidence that the sex of an unfamiliar person can influence canine behavior. To determine whether sex of an unfamiliar walker might influence the behavior of dogs at an animal shelter, we observed 100 dogs during leash walks and recorded all occurrences of scent-marking behaviors. Male dogs urinated at higher rates when walked by unfamiliar women than when walked by unfamiliar men. Female dogs urinated at similar rates when walked by unfamiliar men and unfamiliar women. Sex of walker also influenced urinary posture in male dogs. Both male and female dogs were more likely to defecate when walked by unfamiliar women than when walked by unfamiliar men. Based on our findings, and those of others, we suggest that the sex of all observers and handlers be reported in behavioral studies of dogs and considered in behavioral evaluations at animal shelters, where results can impact whether or not a dog is made available for adoption.

**Abstract:**

Interactions with humans influence the behavior and physiology of other animals, and the response can vary with sex and familiarity. Dogs in animal shelters face challenging conditions and although contact with humans typically reduces stress and behaviors associated with stress, evidence indicates that shelter dogs react differently to unfamiliar men and women. Given that some aspects of canine scent-marking behavior change under fearful conditions, we examined whether sex of an unfamiliar walker would influence scent-marking behavior of 100 shelter dogs during leash walks. Male dogs urinated at higher rates when walked by unfamiliar women than when walked by unfamiliar men; female dogs urinated at similar rates when walked by unfamiliar women and unfamiliar men. Sex of walker influenced urinary posture in male dogs, but not in female dogs. Both male and female dogs were more likely to defecate when walked by unfamiliar women than by unfamiliar men. Based on our findings that shelter dogs behave differently in the presence of unfamiliar men and women, we suggest that researchers conducting behavioral studies of dogs record, consider in analyses, and report the sex of observers and handlers as standard practice. We also recommend recording the sex of shelter staff present at behavioral evaluations because the results of these evaluations can impact dog welfare.

## 1. Introduction

Human presence and handling can affect the behavior and physiology of other animals, including species living in the wild [1,2,3,4] and in captive settings, such as zoos [5,6], farms [7,8,9], and research laboratories [10]. Such effects often depend on the number of people present as well as their distance, behavior, and familiarity [11,12,13,14,15]. Additionally, human physical characteristics, including sex and age, can influence the behavior and physiology of other animals [10,16]. Laboratory rats and mice discriminate human sex using olfactory stimuli [10] and free-ranging elephants discriminate human sex and age using acoustic cues in voices [16]. In many interactions between humans and other animals, humans are perceived as either predators or at least as something to be feared [16,17], thus, human presence often causes increased vigilance, avoidance, and stress.

Domestic dogs present a somewhat special case in which contact with humans typically reduces both stress and the performance of behaviors associated with stress [18,19,20,21,22,23,24,25,26]. This has been shown in animal shelters where dogs experience challenging conditions, such as isolation, lack of control, and exposure to unfamiliar people, dogs, and surroundings. For shelter dogs, various forms of physical contact with humans (e.g., petting, massaging, and grooming) and different types of interactions with humans (e.g., walks, play sessions, training sessions, and simply having a person sit passively in the same enclosure), have been shown to reduce physiological measures of stress [18,19,20], produce favorable changes in behavior [21], or both [22,23,24,25,26].

Despite the general pattern that human contact has positive effects on shelter dogs, there is evidence that dogs respond differently to men and women. Shelter dogs enrolled in a human interaction program improved in their sociability toward unfamiliar women but not toward unfamiliar men [26]. When an unfamiliar man or woman stood in front of cages for a few minutes, shelter dogs decreased to a greater extent the time they spent barking and looking at the person when the unfamiliar person was female [27]. An initial report that shelter dogs petted by females had lower cortisol levels than those petted by males [28] was later found to reflect subtle differences in petting techniques of males and females: when men and women received specific training to standardize petting techniques, male and female petters reduced cortisol levels to similar degrees [20,29]. Differential responses to men and women also have been documented for dogs in settings other than animal shelters. For example, dogs in a guide dog training program made more frequent contact with unfamiliar women than with unfamiliar men [30] and during agility competitions, dogs with male handlers experienced greater increases in cortisol than did dogs with female handlers [31]. Finally, from a study in a commercial kennel, male dogs spent less time near an unfamiliar man than an unfamiliar woman, whereas female dogs spent equal amounts of time near an unfamiliar man and an unfamiliar woman; a similar pattern occurred for the frequency of direct body contact [32]. Although the responses of dogs to male and female humans have been studied in diverse settings and ways, we could find no information on how sex of an unfamiliar walker might influence the behavior of dogs during leash walks.

In the present study, we examined whether sex of an unfamiliar walker influenced scent-marking behavior of mature male and female shelter dogs during walks on a leash. Leash walking is commonly used by shelters to provide dogs with opportunities to exercise, socialize with humans, and perform species-typical behaviors, such as sniffing and urine-marking. At least one aspect of canine scent-marking behavior is sensitive to fearful or stressful conditions: adult male dogs that used the raised-leg urinary posture typical of mature males temporarily reverted in fearful situations to using the juvenile lean-forward posture in which all four feet remain on the ground [33,34]. Consistent with this finding, we reported that the percent of urinations in which adult male dogs used the raised-leg posture was lower in our study shelter (73%; [35]) than reported for mature male dogs living under other conditions (94%–97%; [36,37,38,39]). We found a similar effect for female dogs: 6% of urinations by adult females involved raising a hindlimb at our study shelter [35] compared with 19%–37% for adult female dogs living under other conditions [36,37,38,39]. These observations suggest that monitoring scent-marking behavior of dogs during walks might be a useful way to assess how shelter dogs respond to the sex of an unfamiliar walker. Given that dogs generally respond less favorably to unfamiliar men than unfamiliar women [26,27,30] and that this response can be stronger in male dogs [32], we predicted that the frequency of scent marking behaviors would be lower when dogs were walked by an unfamiliar male than by an unfamiliar female, and that such reductions would be more dramatic in male dogs than in female dogs. We predicted reductions in scent marking behaviors of mature dogs walked by unfamiliar men because mature male dogs reverted to using the juvenile urinary posture in fearful situations [33,34] and less frequent urination, defecation, and ground scratching represent the pattern of scent marking shown by juvenile shelter dogs during leash walks [40]. Finally, we predicted that dogs walked by an unfamiliar male would be more likely to use postures in which all feet remain on the ground (i.e., the lean-forward posture in males and the squat posture in females).

## 2. Materials and Methods

The data presented here were collected at the Tompkins County SPCA in Ithaca NY, USA, between September 2017 and December 2019, as part of a long-term research program on scent-marking behavior of shelter dogs. Tompkins County SPCA is a no-kill shelter with open-admission and scheduled intake. The shelter has very active volunteer programs for both cats and dogs. Dog volunteers must be at least 18 years old and can be either canine companions or dog walkers. Canine companions help socialize and train dogs in their cubicles, sit with them, and pet and groom them. Dog walkers take the dogs out for walks or to a large outdoor enclosure (Section 2.1). As time permits, volunteer dog walkers sometimes engage in canine companion activities as well. A one-time snapshot of dog volunteers at the end of our study showed that 71% (58/82) were women and 29% (24/82) were men. Although the numbers of male and female staff members are complicated by the variation in the extent of direct interaction with the dogs and the needs of individual dogs (e.g., some dogs may have extensive interactions with Medical Staff, whereas others have much less), staff was also female-biased in most positions over the course of our study (e.g., Animal Care Technicians, 10 females and one or two males; Medical Staff, all females except for one male intern in the past 6 months; Adoptions, Intake, and Behavior Program, approximately equal number of females and males).

### 2.1. Dogs and Housing

We observed 100 dogs (57 males and 43 females) that were at least one year old (Mean *± SD*, 4.4 ± 3.4 years; range, 1–17 years). Housing and care of dogs have been described elsewhere [41], thus, we provide a brief description here. Most dogs in our study were mixed breeds. We did not have access to DNA analyses or pedigrees; thus, the number of purebred dogs is unknown. Dogs were either surrendered by owners (*n* = 44), transferred from other shelters (*n* = 24), picked up as strays (*n* = 18), or returned by adopters (*n* = 12); two dogs were seized by animal control officers. All dogs received veterinary care at intake (e.g., vaccinations, flea control, fecal exam and deworming, heartworm test, and any additional diagnostic tests deemed necessary). Dogs without a microchip received one. Screening blood work, including complete blood count/chemistry profile, was routinely run for older dogs. If owners provided information about urinary issues at the time of surrendering their dog to the shelter or if symptoms of disease were observed in the shelter (e.g., frequent urination), then urinalysis was performed for dogs of any age. We excluded from our study dogs with known medical issues. About 3 days after intake, dogs underwent behavioral evaluation by Behavior Program staff [42,43]. All dogs had received veterinary care, undergone behavioral evaluation, and were on the adoption floor by the time we walked them. Dogs on the adoption floor wore buckle or martingale collars and were individually housed in one of 13 cubicles (from 5.2 m^2^ to 7.3 m^2^). Each dog had a water bowl, raised bed with blanket, and toys. Staff fed dogs between 08:00 and 09:00 h and between 15:00 and 16:00 h; additionally, a pre-measured bag of small treats was available for each dog each day. Shelter staff or volunteers either walked dogs or brought them to a large outdoor enclosure several times a day. Each day, the start time and end time of each walk or time in the outdoor enclosure were recorded on a dry erase board in the dog wing.

At the shelter, most dogs are spayed or neutered before placement on the adoption floor; all are spayed or neutered before adoption. In research previously conducted at this shelter, one of us (B.M.) found that rates of urination during walks decreased after castration in males but did not change after spaying in females (within-dog study; [44]). Similarly, intact males urinated at higher rates than castrated males, but intact and spayed females urinated at similar rates (between-dog study; [44]). Gonadectomy did not influence likelihood of defecation or ground scratching during walks in either males or females (between-dog study; [44]). Given the effect of reproductive condition on the rate of urination in male dogs, it was essential that we control for reproductive condition within each male dog when walked by male versus female walkers. Of the 57 male dogs that we observed, 56 were neutered for all of their walks and one was intact for all of his walks. Of the 43 female dogs that we observed, 36 were spayed for all of their observations, four were intact for all of their observations, and three were intact for some observations and spayed for others.

### 2.2. Behavioral Observations

Behavioral observations occurred during walks, which began on shelter grounds and continued into a large field across the street (16.6 ha; 42°28’20”N, 76°26’22”W). The field was bordered by a creek, forest, and other fields of very tall grass; the substrate where we walked was mostly grass, which was occasionally mowed in spring and summer. All procedures were carried out under protocol 2012-0150, which was approved by Cornell University’s Institutional Animal Care and Use Committee.

Over the course of the study, five different walkers (two females, B.M. and D.O., and three males, K.F., L.U., and J.C.) conducted behavioral observations during individual first walks of dogs (i.e., only the walker was present with the dog and this was the first time that person had walked the dog). All walkers had extensive experience handling and observing dogs on walks, gained via research activities at the shelter, long-term dog-walking as a volunteer at the shelter, or independent employment as a dog walker. Individual walkers conducted observations between 12:00 and 17:00 h, typically once or twice a week, on days that were convenient for them. All dogs included in the data set were individually walked by at least one male and one female walker (one male walker and one female walker: 50 dogs; one male walker and two female walkers: 30 dogs; two male walkers and one female walker: 10 dogs; two male walkers and two female walkers: 10 dogs). Dogs were adopted throughout our study, which is why the number of times a given dog was walked varied from two (one male walker and one female walker) to four (two male walkers and two female walkers). Records of specific staff or volunteers who had walked each dog prior to our walks were not available.

On each walking day, a walker checked the dry erase board in the dog wing and selected dogs he or she had never walked before and that had not been outside for at least 2 h. Scheduled dog walking shifts at the shelter occur at 12:00, 14:30, and 17:00 h, thus, dogs included in our study were walked approximately 2–3 h after their previous walk. Once a team member had walked a specific dog for the first time, B.M. alerted other team members to prioritize that dog for walking. We used leashes and harnesses provided by the shelter; staff had previously fitted each dog with an appropriate harness (either a PetSafe Easy Walk Harness, Radio Systems Corporation, Knoxville, TN, USA or a Zack and Zoey Nylon Pet Harness, Pet Any Way LLC, model US2395 14 99) and placed the harness and a cloth lead (at least 1.8 m long) on a hook outside the dog’s cubicle. Upon entering a dog’s cubicle, each walker harnessed the dog, attached the lead and led the dog out of the shelter. Behavioral observations began once the dog was outside and lasted for 20 min, during which time we let dogs determine the pace of the walk (dogs were not kept in a heel position). Per shelter policy, dogs were not allowed to interact with other dogs during walks. We verbally recorded behavioral observations using our cell phones (e.g., the voice memo app on an iPhone 7, model MN9G2LL/A, Apple Inc., Cupertino, CA, USA). We recorded each urination, defecation, and occurrence of ground scratching (backward scraping of the ground with the front feet, hind feet, or both performed by some dogs after urination or defecation). For each urination, we also recorded the posture used (female postures: squat, used by juvenile females and most adult females, and squat raise, used by some adult females; male postures: lean forward, used by adult males under fearful conditions and juvenile males, and raised leg, typical posture for adult males; [39,45]). At the end of walks, we returned dogs to their cubicles and retrieved relevant information from shelter records (e.g., dog identification number, intake date, source, and age). We used the intake date to calculate the number of days each dog had been at the shelter at the time of each of its walks with us (= time at shelter); for dogs that were adopted and returned to shelter, time at shelter was left blank. (Note that this meant that the 12 returned dogs were dropped from analyses, which included time at shelter as a main effect and the interaction between time at shelter and walker sex; see Section 2.3). Each dog was photographed. We transferred data from verbal recordings to paper check sheets within hours of walks and scanned each check sheet as a .pdf file.

### 2.3. Statistical Analyses

A linear mixed model was used to model the rate of urination (total number of urinations/20 min) and a generalized linear mixed model with a binominal distribution and logit link was used to model defecation (yes/no) and ground scratching (yes/no) during walks. We used a generalized estimating equation (GEE) to model the predominant urinary posture. We defined the predominant posture as the posture used most frequently during a walk; ties were recorded as such. We coded males whose predominant posture was either the raised leg or a tie between the raised leg and the lean forward as one; those whose predominant posture was the lean forward as zero (i.e., not involving a raised hindlimb). Similarly, we coded females whose predominant posture was the squat raise or a tie between the squat raise and squat as one; females whose predominant posture was the squat were coded as zero (again, not involving a raised hindlimb). All models were initially fit with the fixed effects of the dog’s sex and the walker’s sex, and the interaction between the dog’s sex and the walker’s sex. Time at shelter was included as a main effect and interacted with walker’s sex. In all of the mixed models, we included the dog’s ID as a random effect; in the GEE model, we treated the dog’s ID as a cluster effect with an unstructured covariance matrix. Reduced models were obtained by removing interactions that were not significant (except for the interaction between dog sex and walker sex, which was retained in models due to research interest), and then removing the main effects that were not significant. For the rate of urination, we used Cohen’s d to calculate the effect size. Data were analyzed using either JMP Pro 12 (2015. SAS Institute, Cary, NC, USA) or R, version 3.6.2 (R Foundation for Statistical Computing, Vienna, Austria).

## 3. Results

Descriptive statistics for the three scent-marking behaviors and time at shelter are shown in Table 1. The statistics in Table 1 are meant to provide a general overview of the raw behavioral data collected and the length of time dogs had been at the shelter at the time of their walks.

The results that follow are from the reduced models. The results from the full models are provided as Supplementary Material.

### 3.1. Urination Rate

**Figure 1:**
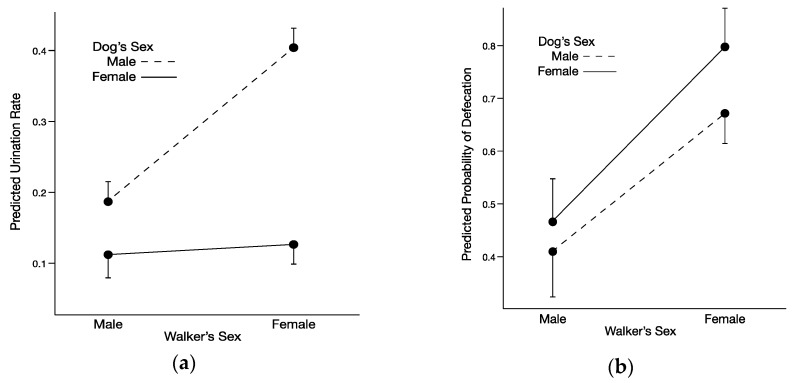
Scent-marking behaviors of shelter dogs in relation to sex of dog and sex of walker. (**a**) Predicted rates of urination by male and female dogs when walked by male or female walkers. (**b**) Predicted probabilities of defecation by male and female dogs when walked by male or female walkers. Walks were 20 min in duration.

Of the six male dogs that did not urinate during their walks, five did not urinate when walked by a male walker but did urinate when walked by a female walker, and the remaining dog did not urinate when walked by either male or female walkers. One female dog did not urinate when walked by a male walker but did urinate when walked by a female walker. Dogs that did not urinate during walks were included in analyses. We found a significant interaction between dog sex and walker sex for rate of urination (total number of urinations/20 min; Table 2). Male dogs urinated at higher rates when walked by female walkers than when walked by male walkers (d = 0.87); in contrast, female dogs urinated at similar rates when walked by male walkers and female walkers (d = 0.36; Figure 1a). Additionally, sex differences in rates of urination (urination rates of male dogs > urination rates of female dogs) were apparent with female walkers but not with male walkers (Figure 1a). The main effect for time at shelter also was significant (Table 2).

### 3.2. Likelihood of Defecation

We found a significant main effect of walker sex on likelihood that a dog would defecate during a walk (Table 3). A dog had a 0.441 probability of defecating with a male walker and a 0.740 probability of defecating with a female walker. We did not find a significant interaction between dog sex and walker sex for the likelihood that a dog would defecate during a walk (Table 3; Figure 1b). The odds that a male dog will defecate with a female walker are 2.9 times larger than with a male walker (*p* = 0.013). The odds that a female dog will defecate with a female walker are 4.5 times larger than with a male walker (*p* = 0.004).

### 3.3. Likelihood of Ground Scratching

There were no significant predictors of ground scratching during a walk (Table 4).

### 3.4. Urinary Postures

For male dogs, the raw data revealed the following percentages of walks in which the lean forward was the predominant urinary posture (i.e., all limbs remain on the ground when urinating): when walked by male walkers, 20.6%; when walked by female walkers, 13.2%. For female dogs, the raw data revealed the following percentages of walks in which the squat was the predominant urinary posture (again, all limbs remain on the ground when urinating): when walked by male walkers, 92.0%; when walked by female walkers, 95.1%. We found a significant interaction between dog sex and walker sex (Table 5). Male dogs were more likely to use the lean-forward posture when walked by a male walker than when walked by a female walker; in contrast, the likelihood of female dogs using the squat posture did not differ when walked by male walkers or female walkers. The odds of a male dog using the lean-forward posture as its predominant posture were 1.9 times greater when walked by a male walker (predicted probability of 0.222) than when walked by a female walker (predicted probability of 0.127; *p* = 0.052). Finally, the predicted probability of a female dog using the squat posture as its predominant posture was 0.922 when walked by a male walker, which did not differ from the predicted probability of a female dog using the squat posture as its predominant posture when walked by a female walker (0.941; *p* = 0.33).

## 4. Discussion

We found that two scent-marking behaviors of shelter dogs—urination (as measured by urination rate) and defecation (as measured by occurrence during a walk)—were influenced by the sex of an unfamiliar walker. Ground scratching, also measured by occurrence during a walk, was not affected by walker sex. The predominant urinary posture during a walk also was affected by walker sex. Only urination rate was affected by time spent at the shelter: rate of urination slightly declined with increasing time spent at the shelter.

In the case of urination rate, the effects of walker sex varied with sex of dog. Male dogs urinated at higher rates when walked by unfamiliar women than when walked by unfamiliar men, whereas female dogs urinated at similar rates when walked by unfamiliar women and unfamiliar men. In fact, the well-established pattern of higher rates of urination by mature male dogs than mature female dogs [37,39,40,46] was present with female walkers but disappeared with male walkers in our study (Figure 1a). For the predominant urinary posture, male dogs were more likely to use the lean-forward posture when walked by unfamiliar men than when walked by unfamiliar women. The urinary posture of female dogs did not differ when walked by unfamiliar men and unfamiliar women. These findings for urination rate and predominant urinary posture support our predictions and are similar to results reported for dogs responding to the presence of either an unfamiliar man or an unfamiliar woman in a commercial kennel setting. Lore and Eisenberg [32] found that male dogs spent less time near an unfamiliar man than an unfamiliar woman, whereas female dogs did not differ in this regard; the authors found a similar pattern for direct body contact with an unfamiliar man and an unfamiliar woman. For the likelihood that a dog would defecate during a walk, we found a main effect of sex of walker but no interaction between sex of walker and sex of dog: both male dogs and female dogs were more likely to defecate when walked by an unfamiliar woman than when walked by an unfamiliar man (Figure 1b). Wells and Hepper [27] found a similar pattern when either an unfamiliar man or an unfamiliar woman stood at the front of cages for a few minutes: both male and female shelter dogs decreased to a greater extent time spent barking and looking at the person when the unfamiliar person was female. In summary, depending on the particular category of behavior used to assess response of dogs to unfamiliar people, the presence of an unfamiliar man can either uniquely affect male dogs (time spent in proximity, time spent in direct contact, rate of urine marking, and predominant urinary posture), affect male and female dogs in a similar manner (time spent barking, time spent looking, and likelihood of defecation), or have no significant effect on either male or female dogs (ground scratching). Ground scratching is performed by a minority of shelter dogs (present study; [40]). Cafazzo et al. [37] studied members of a feral dog pack and found that high ranking individuals ground scratched more frequently than low ranking ones. Perhaps dogs that ground scratch are more confident, which might explain our failure to find an effect of sex of walker for this particular scent-marking behavior.

Our finding that rate of urination declined with increasing time spent at the shelter was unexpected; although the size of this effect was very small, it nonetheless was significant. In a previous study of scent-marking behavior during first walks of dogs at the same shelter, we found that time spent at the shelter did not influence urinary behavior (frequency of urination or percent of urinations directed at targets in the environment) or likelihood of defecation or ground scratching [40]. In a subsequent study in which many dogs were walked multiple times [47], we found that time spent at the shelter positively influenced rate of urination, percent of directed urinations, and likelihood of defecation (ground scratching was not studied). In light of our findings for first walks [40], we interpreted the positive influence of time at shelter in our second study [47] as having resulted from our inclusion of multiple walks on individual dogs, and suggested that the positive influence of time spent at the shelter on marking behavior could reflect dogs becoming more familiar with their surroundings and routine, as well as with us [47]. For these two earlier studies [40,47], there were no single male walkers or single female walkers; we always had two people on each walk, one student (male or female) and B.M. (one person walked the dog and the other recorded behavioral observations). These methodological differences make our current finding regarding urination rate and time at shelter challenging to interpret. One possible interpretation is that more timid dogs are characterized by longer stays at the shelter as well as lower rates of urination.

We did not determine the precise stimuli by which shelter dogs discriminated sex of an unfamiliar walker. Potential stimuli include olfactory, visual, auditory, and tactile/handling differences between male and female walkers. With respect to the latter, subtle differences in petting techniques of males and females appeared responsible for an initial report that shelter dogs petted by females had lower cortisol levels than those petted by males [28]. In two subsequent studies in which men and women received specific training to standardize their petting techniques, male and female petters reduced cortisol levels to the same degree [20,29]. In contrast, androgen-based olfactory cues are used by laboratory mice and rats to discriminate experimenter sex: olfactory cues from men caused a physiological stress response that induced an inability to feel pain in both rodent species [10]. Acoustic cues in voices are used by elephants to discriminate human sex and age: in response to playbacks of either adult male or adult female Maasai voices, members of elephant families were more likely to retreat and exhibit defensive bunching when hearing male voices [16]. It is important to determine which features of unfamiliar men shelter dogs attend to, so that the effectiveness of human interaction during walks, and perhaps other enrichment activities, can be maximized. Additionally, determining whether the observed reactions to unfamiliar male walkers disappear with familiarity would be useful.

Our study focused on scent-marking behaviors of dogs during walks by unfamiliar males and unfamiliar females; we did not measure the physiological responses of dogs to unfamiliar male and female walkers. However, Alberghina et al. [48,49] conducted two studies with shelter dogs to investigate the relationships between scent marking, cortisol, and supervised social exposures with another dog. Social exposures occurred in a fenced area, with both dogs initially on leashes and then eventually off leashes. In the first study, Alberghina et al. [48] found a significant positive relationship between frequency of urine-marking by dogs during social exposures and urinary cortisol-creatinine ratio (C/Cr) measured several hours later and a significant negative relationship between the frequency of defecation during social exposures and C/Cr. In the subsequent study, which differed from the first in some aspects of methodology (e.g., dogs were habituated to muzzles before social exposures in the second study but not in the first), Alberghina et al. [49] found the same patterns with respect to urination and C/Cr and defecation and C/Cr but the results did not reach statistical significance. As suggested by Alberghina et al. [48,49] and Protopopova [50], elevated levels of cortisol could indicate increased arousal and activity, rather than stress in dogs. Results from mammals studied under laboratory conditions suggest a complicated relationship between stress, scent marking, and cortisol. For example, when housed without access to a preferred outdoor cage, common marmosets exhibited elevations in cortisol and increases in scent marking behavior (rubbing scent glands on the substrate) [51]. In contrast, male Mongolian gerbils exhibited elevated cortisol levels but reduced scent marking (rubbing the ventral gland on the substrate) when subjected to social defeat, a stress paradigm in which a male is repeatedly paired with a dominant male conspecific [52]. Thus, elevated cortisol has been associated with both increases and decreases in scent marking behavior in mammals, suggesting the relationship between scent marking, stress, and cortisol requires further study across species and stress paradigms.

A limitation of our study is that age also varied among walkers (female walkers: D.O., 21, B.M., 61; male walkers: J.C., 20, K.F., 22, L.U., 31). Few studies have examined the influence of human age on dog behavior, except in regard to dog bites (e.g., [53]). Although Koda and Shimoju [30] found that dogs enrolled in a guide dog program made more frequent contact with unfamiliar women than unfamiliar men, they found no difference in the frequency with which dogs contacted unfamiliar females who were either between 20 and 40 years old or between 8 and 13 years old. These findings suggest that, at least in the case of unfamiliar females, age might not matter to dogs; Koda and Shimoju [30] did not examine response of dogs to unfamiliar males from different age groups. Dog volunteers at the Tompkins County SPCA ranged from 18 years old to over 70 years old; thus, the dogs in our study likely had some experience interacting with humans of diverse ages before we walked them.

## 5. Conclusions

Given that sex of an unfamiliar human has been shown to affect both the in-kennel behavior of shelter dogs [27] and their behavior outside the kennel during leash walks (present study), we suggest that researchers conducting behavioral observations of shelter dogs (and perhaps dogs generally) record, consider in their analyses, and report the sex of observers/handlers as standard practice. Based on their findings that experimenter sex influenced the behavior and physiology of laboratory mice and rats, Sorge et al. [10] made a similar recommendation for researchers studying any phenomenon in laboratory rodents that could be affected by stress. Our findings might also have implications for canine behavioral evaluations at animal shelters. Such evaluations are usually conducted a few days after intake, when dogs are likely unfamiliar with at least some staff present at these tests. Additionally, behavioral evaluations often include a subtest in which an unfamiliar person knocks on the door and enters the room where testing is taking place (e.g., Stranger Test in the Modified Assess-A-Pet; [42,43]). Shelter dogs have been shown to differentiate sex of an unfamiliar person during behavioral evaluations: Bergamasco et al. [26] found that shelter dogs enrolled in a human interaction enrichment program and behaviorally evaluated several times over a period of weeks improved in their responses to unfamiliar females but not to unfamiliar males. Thus, sex of the unfamiliar person and perhaps sex of the evaluator/handler and scribe, could potentially influence results of canine behavioral evaluations, which might then affect dog welfare by influencing whether or not a dog is made available for adoption.

## Figures and Tables

**Table 1:** Descriptive statistics (Mean ± *SD*) for rate of urination by male and female dogs during a 20-min walk by either male or female walkers, along with time at shelter. Additionally, percentage of walks in which dogs defecated or ground scratched are shown.

Dog’s Sex	Walker’s Sex	Urination Rate ^1^	% Walks with Defecation	% Walks with Ground Scratching	Time at Shelter (Days)
Male	Male	0.20 ± 0.15	43.5	37.7	14.8 ± 8.0
	Female	0.40 ± 0.28	61.5	34.6	13.6 ± 9.0
Female	Male	0.10 ± 0.07	49.0	27.5	13.5 ± 6.4
	Female	0.14 ± 0.10	72.6	24.2	13.1 ± 7.1

^1^ Total number of urinations/20 min.

**Table 2:** Effects of sex of dog, sex of walker, and time at shelter on rate of urination per min by dogs during a 20-min walk.

Parameter	Estimate	*SE*	*df*	*t* Value	*p*
Intercept	0.243	0.037	173.464	6.638	<0.001
Dog’s sex					
Female	−0.078	0.041	136.412	−1.888	0.06
Male					
Walker’s sex					
Female	0.218	0.022	139.614	9.644	<0.001
Male					
Time at shelter	−0.004	0.002	206.940	−2.286	0.02
Dog’s sex × Walker’s sex					
Female × Female	−0.203	0.037	137.708	−5.548	<0.001
Female × Male					
Male × Female					
Male × Male					

**Table 3:** Effects of sex of dog and sex of walker on likelihood of defecation by dogs during a 20-min walk.

Parameter	Estimate	*SE*	*z* Value	*p*
Intercept	−0.360	0.364	−0.988	0.32
Dog’s sex				
Female	0.241	0.555	0.435	0.66
Male				
Walker’s sex				
Female	1.073	0.431	2.491	0.013
Male				
Dog’s sex × Walker’s sex				
Female × Female	0.426	0.646	0.659	0.51
Female × Male				
Male × Female				
Male × Male				

**Table 4:** Effects of sex of dog and sex of walker on likelihood of ground scratching by dogs during a 20-min walk.

Parameter	Estimate	*SE*	*z* Value	*p*
Intercept	−1.279	0.699	−1.83	0.07
Dog’s sex				
Female	−1.317	0.989	−1.33	0.18
Male				
Walker’s sex				
Female	−0.214	0.532	−0.40	0.69
Male				
Dog’s sex × Walker’s sex				
Female × Female	0.081	0.821	0.10	0.92
Female × Male				
Male × Female				
Male × Male				

**Table 5:** Effects of sex of dog and sex of walker on the likelihood of dogs having a predominant urinary posture in which all limbs remain on the ground (i.e., lean-forward posture in males and squat posture in females).

Parameter	Estimate	*SE*	*z* Value	*p*
Intercept	−1.268	0.310	−4.09	<0.001
Dog’s sex				
Female	3.737	0.576	6.48	<0.001
Male				
Walker’s sex				
Female	−0.658	0.338	−1.95	0.052
Male				
Dog’s sex × Walker’s sex				
Female × Female	0.962	0.460	2.09	0.036
Female × Male				
Male × Female				
Male × Male				

## Supplementary Materials

The following are available online at https://www.mdpi.com/2076-2615/10/4/632/s1, Table S1: Effects of sex of dog, sex of walker, and time at shelter on the rate of urination per min by dogs during a 20-min walk. Results are from the full model, Table S2: Effects of sex of dog and sex of walker on the likelihood of defecation by dogs during a 20-min walk. Results are from the full model. Table S3: Effects of sex of dog and sex of walker on the likelihood of ground scratching by dogs during a 20-min walk. Results are from the full model. Table S4: Effects of sex of dog and sex of walker on the likelihood of dogs having a predominant urinary posture in which all limbs remain on the ground (i.e., lean-forward posture in males and squat posture in females). Results are from the full model.
