# Supplementary material for: Sex of Walker Influences Scent-marking Behavior of Shelter Dogs
Source: Animals (Basel). 2020 Apr 7;10(4):632. doi: 10.3390/ani10040632 (PMC7222742; doi:10.3390/ani10040632)
Supplement: Supplementary file 1 [file animals-10-00632-s001.pdf]

Table S1. Effects of sex of dog, sex of walker, and time at shelter on rate of urination per min by dogs during a 20-min walk. Results are from the full model.

Parameter	Estimate	*SE*	*df*	***t*** **value**	***P***
Intercept	0.210	0.042	206.536	4.975	< 0.001
Dog's sex					
Female	-0.074	0.042	138.301	-1.781	0.08
Male					
Walker's sex					
Female	0.273	0.041	140.156	6.727	< 0.001
Male					
Time at shelter	-0.002	0.002	212.926	-0.783	0.44
Dog's sex X Walker's sex					
Female X Female	-0.207	0.037	136.884	-5.628	< 0.001
Female X Male					
Male X Female					
Male X Male					
Walker's sex X Time at shelter					
Female X Time at shelter	-0.004	0.002	141.233	-1.636	0.10
Male X Time at shelter					

Table S2. Effects of sex of dog and sex of walker on likelihood of defecation by dogs during a 20-min walk. Results are from the full model.

Parameter	Estimate	*SE*	***z*** **value**	***P***
Intercept	-0.006	0.715	-0.009	0.99
Dog's sex				
Female	0.313	0.660	0.474	0.64
Male				
Walker's sex				
Female	1.260	0.805	1.565	0.12
Male				
Time at shelter	-0.028	0.041	-0.697	0.49
Dog's sex X Walker's sex				
Female X Female	0.731	0.767	0.953	0.34
Female X Male				
Male X Female				
Male X Male				
Walker's sex X Time at shelter				
Female X Time at shelter	-0.015	0.046	-0.315	0.75
Male X Time at shelter				

Table S3. Effects of sex of dog and sex of walker on likelihood of ground scratching by dogs during a 20-min walk. Results are from the full model.

Parameter	Estimate	*SE*	*z* value	*P*
Intercept	-2.859	1.527	-1.87	0.06
Dog's sex				
Female	-1.567	1.310	-1.96	0.23
Male				
Walker's sex				
Female	0.401	1.081	0.37	0.71
Male				
Time at shelter	0.071	0.064	1.11	0.26
Dog's sex X Walker's sex				
Female X Female	0.637	1.014	0.63	0.53
Female X Male				
Male X Female				
Male X Male				
Walker's sex X Time at shelter				
Female X Time at shelter	-0.025	0.060	-0.42	0.67
Male X Time at shelter				

Table S4. Effects of sex of dog and sex of walker on likelihood of dogs having a predominant urinary posture in which all limbs remain on the ground (i.e., lean-forward posture in males and squat posture in females). Results are from the full model.

Parameter	Estimate	*SE*	*z* value	*P*
Intercept	-1.746	0.517	-3.38	< 0.001
Dog's sex				
Female	4.101	0.692	5.92	< 0.001
Male				
Walker's sex				
Female	-0.303	0.572	-0.53	0.60
Male				
Time at shelter	0.034	0.030	1.16	0.25
Dog's sex X Walker's sex				
Female X Female	0.710	0.545	1.30	0.19
Female X Male				
Male X Female				
Male X Male				
Walker's sex X Time at shelter				
Female X Time at shelter	-0.022	0.030	-0.72	0.47
Male X Time at shelter				
